# STAC: Spatial-Temporal Attention on Compensation Information for Activity Recognition in FPV

**DOI:** 10.3390/s21041106

**Published:** 2021-02-05

**Authors:** Yue Zhang, Shengli Sun, Linjian Lei, Huikai Liu, Hui Xie

**Affiliations:** 1 Shanghai Institute of Technical Physics, Chinese Academy of Sciences, Shanghai 200083, China; zhangyue8@mail.sitp.ac.cn (Y.Z.); leilj@shanghaitech.edu.cn (L.L.); liuhuikai@mail.sitp.ac.cn (H.L.); xiehui@mail.sitp.ac.cn (H.X.); 2 School of Electronic, Electrical and Communication Engineering, University of Chinese Academy of Sciences, Beijing 100049, China; 3 Key Laboratory of Intelligent Infrared Perception, Chinese Academy of Sciences, Shanghai 200083, China; 4 School of Information Science and Technology, ShanghaiTech University, Shanghai 200083, China

**Keywords:** egocentric video analysis, location-aware attention, compensation information, fine-grained activity recognition

## Abstract

Egocentric activity recognition in first-person video (FPV) requires fine-grained matching of the camera wearer’s action and the objects being operated. The traditional method used for third-person action recognition does not suffice because of (1) the background ego-noise introduced by the unstructured movement of the wearable devices caused by body movement; (2) the small-sized and fine-grained objects with single scale in FPV. Size compensation is performed to augment the data. It generates a multi-scale set of regions, including multi-size objects, leading to superior performance. We compensate for the optical flow to eliminate the camera noise in motion. We developed a novel two-stream convolutional neural network-recurrent attention neural network (CNN-RAN) architecture: spatial temporal attention on compensation information (STAC), able to generate generic descriptors under weak supervision and focus on the locations of activated objects and the capture of effective motion. We encode the RGB features using a spatial location-aware attention mechanism to guide the representation of visual features. Similar location-aware channel attention is applied to the temporal stream in the form of stacked optical flow to implicitly select the relevant frames and pay attention to where the action occurs. The two streams are complementary since one is object-centric and the other focuses on the motion. We conducted extensive ablation analysis to validate the complementarity and effectiveness of our STAC model qualitatively and quantitatively. It achieved state-of-the-art performance on two egocentric datasets.

## 1. Introduction

Understanding human activities from videos is a widely studied problem in computer vision. The emergence of smart wearable devices has spawned rich egocentric video studies. First-person video (FPV) [1] obtained through wearable devices mainly records the daily activities of the actor, which has promoted the extensive establishment of daily life datasets. Georgia Institute of Technology has successively proposed GTEA [2,3], GTEA_GAZE [4], EGTEA_GAZE+ [5] and other video datasets containing daily activities in the kitchen. Cruz et al. [6] proposed a dataset of lifelogging: Ego-Daily consists of 50,000 annotated images with 10 different subjects doing five different daily activities, including cycling, eating, office, running, meal preparation, etc. They also presented a model to detect first-person hands. Alahari K. et al. [7] built a dataset which includes pairs of first-person and third-person videos providing links from the first-person perspective to extensive third-person data on the Internet to learn the joint representation of the two perspective videos. At present, the most representative and challenging ultra-large-scale first-person dataset Epic-Kitchens [8] defines three challenges in FPV: activity recognition, object detection and activity prediction. We address the challenging problem of egocentric fine-grained activity recognition in a short-term video lasting a few seconds.

In this task, hands are the performers of actions and the objects being manipulated are also worthy of attention. This is more challenging than action recognition only, which is necessary to identify the actions and the activated objects in pairs simultaneously, such as “pour_water, open_door, fold_bread”, etc. The particularities of the wearable device make the information analysis more difficult: (1) The ego-noise introduced by the wearer’s head movement brings sharp changes to the frames, making the motion encoding more complicated. (2) In addition, a variety of small and detailed objects also make it difficult to distinguish between activated objects and those escaping our attention. This may require fine-grained, frame-level annotations, which are very expensive and impractical in large-scale daily logging. (3) Developing the relevant representations of ongoing actions and objects is the core issue in this task. Therefore, there has been a large amount of literature [9,10,11] dedicated to third-person surveillance video understanding, and these action recognition techniques have been successfully applied to first-person video analysis. However, these works are mainly designed for third-person action recognition, and egocentric activity recognition requires simultaneous recognition of action and fine-grained objects.

**Figure 1:**
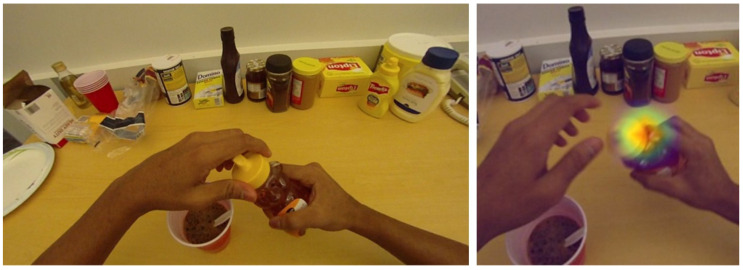
The actor’s attention is focused on what he is doing. The area of the operator’s attention is usually the focus of the person’s vision, and thus can be used as prior information to define the video activity.

Earlier works mainly take the matter from egocentric cues [3,12,13]. In recent years, a large number of video action recognition works, whether in third-person or in first-person, have tended to focus on appearance expression and temporal modeling at the same time. The standard two streams [9] or their deformation [6], and even multi-stream [14] networks integrate various information from the original video. Inspired by this, we developed a “two-stream CNN-RAN” framework called STAC aiming at paired matching recognition of verbs and nouns. However, where does attention come from? We can observe in Figure 1 that the focus of the actor’s attention when performing the task tends to be at the center of the image. We use the class activation map (CAM) proposed by Zhou et al. [15] to generate a heat map from our model to visualize the recognition results and mark the focused intensity. The area covered by the heat map can be used as prior information to define the behavior. Therefore, this work mainly explores the guiding role of attention in fine-grained recognition tasks to localize the relevant spatiotemporal regions.

Attention mechanisms have been widely used. Since Google DeepMind [16] proposed to deploy attention around recurrent neural networks, the structure of Attention-RNN has gradually been applied to first-person video understanding [17,18,19] and achieved excellent performance. In the literature [20], a novel recurrent neural unit LSTA (Long-Short Term Attention) has been proposed. They studied the shortcomings of Attention-LSTM and modified the conventional recurrent neural unit LSTM with a built-in attention component to suppress irrelevant information and smoothly track the potential activation features from RGB frames. In our work, we use the recurrent attention neural (RAN) unit LSTA as a recurrent location-aware tracker and deploy it to the appearance stream and temporal stream respectively.

**Figure 2:**
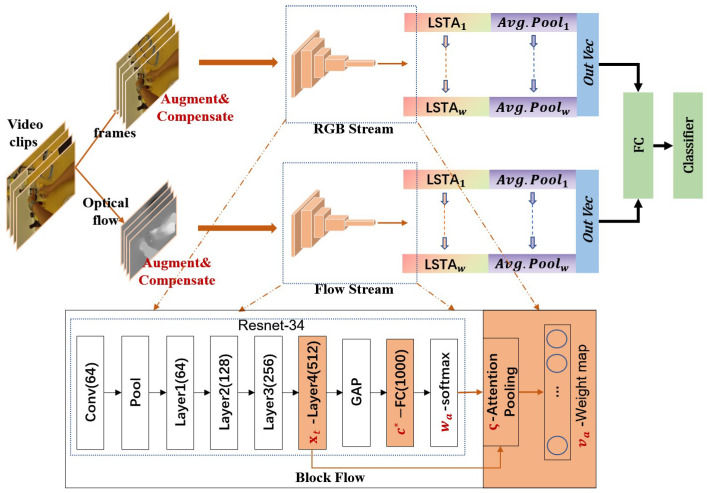
The proposed CNN-RAN model STAC. Our framework consists of SAC (spatial attention on compensation) and TAC (temporal attention on compensation). The nets compensate for the single-scale data and small objects, and the camera motion in the optical flow respectively. Both of the two nets deploy location-aware attention LSTA on the global feature to generate generic attention maps for long-term tracking and guiding egocentric activity recognition. The bottom diagram (block flow) is the detailed interpretation of the feature extractor and the attention pooling of recurrent neural attention LSTA. It uses the idea of global average pooling (GAP) from class activation map (CAM) to get a location-aware weight map as the input of recurrent neural cells to maintain the location information.

The objective of this article is to address the problem of fine-grained activity recognition in FPV by developing an architecture that is able to generate generic descriptors of localization of spatial activation and temporal motion. Section 3 describes the proposed method and the overall framework is shown in Figure 2. In particular, Section 3.2 investigates the size compensation and CNN is used to extract the deep features of compensated frames followed by a recurrent location-aware attention mechanism to track the potential activation features; in Section 3.3, we compensate for the ego-noise of the camera introduced by head motion and size compensation. We present a similar structure as RGB stream to build temporal attention in the form of stacked optical flow. The spatial position perception in temporal series is inspired by the fact that not all frames in the video can contribute equally to the task since the short-term video clips record real daily life and are randomly cropped. Our temporal channel attention model is then able to index the location of the keyframes which can define the activity in the time series. Section 4 shows the experimental details. We introduce the datasets and validation methodology in Section 4.1 and the experimental settings in Section 4.2. Section 4.3 demonstrates the effectiveness of each component of our model in detail by conducting extensive ablation analysis. We compare our method with the state-of-the-art in Section 4.4 and discuss the visualization results in detail to prove the superiority of the proposed model in Section 4.5. The contributions of our work are as follows:

- We performed two-stream compensation and prove the effectiveness through a large number of comparative experiments: size compensation to augment the data and strengthen the localization of activation; motion compensation to eliminate the camera noise and capture effective motion.
- We investigated the importance of location-aware attention in fine-grained activity recognition, and designed a two-stream CNN-RAN model STAC to describe the first-person video. Our benchmark framework does a systematic evaluation of the location-aware attention, which proves its ability to locate objects accurately in the appearance stream and the ability to track effective motion in the motion stream.
- We trained our model end-to-end under a weak supervision setting without complex image preprocessing or additional supervision. As a consequence, our model is able to learn generic features from FPV. We conducted extensive ablation analysis and proved the innovativeness and effectiveness of our model through quantitative comparison and qualitative visualization. We achieved state-of-the-art performance on two egocentric datasets with up to 106 activity classes.

## 2. Related Work

### 2.1. Action Recognition

The wide application of computer vision-based action recognition in the fields of intelligent monitoring, human-computer interaction, robotics, elderly care, medical diagnosis, psychology, etc., has led to extensive research and produced a large number of excellent studies [21,22,23]. Among them, [21] addresses the task of complex action recognition (such as “open laptop”) in videos of minute-long temporal patterns. These works are mainly aimed at recognizing human behavior in third-person videos. Earlier methods are mainly based on hand-made features, using body features as descriptions. Action recognition can be described at two levels [24]: (1) holistic representations such as motion energy image (MEI) and motion history image (MHI) proposed by Bobick et al. [25]; (2) local representations such as [26] and [27]. The promising success of CNN in the image field has inspired some studies to propose action recognition CNNs. One of the most popular methods is two-stream CNNs [9], which consists of a spatial CNN that describes the appearance and a temporal CNN that encodes inter-frame motion. It is 3D-CNN [10,22,28] that achieved promising performance later. In addition, the continuity of video frames introduces recurrent neural networks (RNN) that are very good at processing sequences to action recognition [16,29,30,31]. These technologies mainly represent motion patterns and do not involve object recognition of active operations.

### 2.2. Egocentric Activity Recognition

**Ego-Cues:** Different from third-person video analysis, the behaviors in egocentric videos mainly revolve around hand-object interaction. Therefore, hand extracting and object recognition are very important. Some works are devoted to describing the characteristics of egocentric videos. Since hands are the most consistent subjects in FPV, early work suggested complex segmentation and extraction, taking hand features and object segmentation as important egocentric cues [2,3,32]. Cruz et al. [6] proposed to combine pre-trained YOLO and context around hands into a two-stream network to locate the hand position in their Ego-Daily dataset. However, the above work requires manual feature extraction or additional supervision, such as bounding boxes or hand masks. Fathi et al. [12] jointly modeled actions and objects to learn the hierarchical model of activities; Singh et al. [13] jointly encoded egocentric cues, including hand mask, saliency map and camera motion. Li et al. [3] used a wealth of egocentric information, including head motion, hand gesture and gaze information, to encode a set of egocentric features and integrate them with dense trajectory features. Their follow-up work [5] modeled gaze as a stochastic probabilistic variable in a deep network to selectively recognize visual features and thereby recognize the activity. Singh et al. [33] used trajectory aligned features as action descriptors, but their method can only recognize actions and ignores the very important objects in first-person activity recognition.

**Multi-Stream:** Some studies recommend simultaneous encoding of appearance expression and motion optical flow in egocentric activity recognition tasks. The mainstream method commonly used at present is the two-stream method [9], one stream of which describes the appearances of RGB frames, and the other encodes the optical flow. Some works [34,35] take additional egocentric information as the third stream to add privileged information to the two-stream model. These works show that rich self-centered cues can be used as effective features for activity recognition, but complex preprocessing or additional annotations are often required to construct the third stream. Kazakos et al. [34] proposed a novel three-modal model that can capture the audio characteristics when activity occurs. They use the audio information in a specific scene as a third modality and combine the three streams before temporal binding. This model can not only make up for the lack of features when occlusion occurs, but also makes full use of egocentric cues to reduce the impact of environmental noise on activity recognition.

**Attention-RNN:** In FPV, the location of the activity always aligns with the direction of the wearer’s line of sight which tends to be in the center of the image. The attention mechanism can selectively focus on task-related information. In recent years, it has been used in natural language processing [36], speech recognition [37], video caption [38] and achieve excellent performance. Meanwhile, the sequentiality of video inspires studies to use RNNs, especially LSTM because of its efficiency on video expression [39]. In 2014, the Google DeepMind team proposed using the attention mechanism for image classification in the RNN model [16]. Since then, this structure has been widely used to learn detailed spatial or temporal behavior expressions [40,41]. It has recently been successfully used to identify egocentric activities. Sudhakaran et al. [17] used class activation mapping (CAM) [15] as their attention and input it into convLSTM to learn detailed spatial information of objects, and the objects’ locations they dedicate to modeling object position in independent frames, while ignoring the unequal contributions of different frames in temporal sequence. Their subsequent work [20] made a great contribution. They transformed the LSTM and proposed a new recurrent neural unit LSTA. Global average pooling (GAP) [42] was used as a built-in attention component to expand LSTM. In this way, the continuous attention filter generated in the RGB sequence is smoothly tracked. The above two attention mechanisms follow the idea of CAM to ensure that the spatial information of the convolutional features is not lost. In fact, CAM has been proven to have excellent performance in fine-grained recognition [15] for its ability to generate generic localizable deep features. There are few works that use the structure of Attention-RNN in FPV activity recognition. Wang et al. [35] proposed a symbiotic attention mechanism. They normalize the feature of one branch and merge it with another branch to strengthen the communication of information between different branches. Besides, they emphasize the application of privilege information and the associations between verbs and nouns. However, they ignore the propagation of attention weight along with temporal series. Sagar V. et al. [43] proposed a CNN-RNN structure of ResNet50+LSTM that uses a two-stream network. Their work is a concise and effective network, but they did not consider the great effect of the attention mechanism in the description of egocentric video. They only recognize verbs, and our task requires us to recognize combinations of verbs and nouns. In summary, LSTMs have a strong gradient flow which can learn long-term dependence on the attention map obtained from CNN features. To this end, we present a two-stream CNN-RAN structure. It is relatively concise (no manual features or additional supervision need), logical and outstanding.

**Separate branch:** For large datasets such as EGTEA_GAZE+ and EPIC-Kitchens with very large vocabularies, some works use a dual-branch structure to identify verbs and nouns separately. The verb classifier focuses on the ongoing action, while the noun classifier is used to identify the object being manipulated. Wu et al. [44] combine 3D-CNN and a long-term feature bank with object detection features to detect verbs and nouns independently, but ignore the matching relationship between the two. Wang et al. [35] proposed a multi-branch structure. They fine-tuned a pre-trained Faster R-CNN on ego-data and got an object detector, and fused it with a verb detector and noun detector respectively. The symbiotic attention is proposed to enable communication between VerbNet and NounNet. In fact, the abundant additional ego-features are helpful for activity recognition, but whether they are manual features or an end-to-end third stream, strong supervision is required, such as hand masks or object bounding box labeling. Different datasets often have different ego-features, instead of using specific ego-cues from a specific dataset; in this work, the recurrent spatial location-aware attention is embedded in an RGB stream and a motion stream separately to learn egocentric generic features of fine-grained activity. We fused the two streams to get our model STAC and trained it end-to-end in a weakly supervised setting.

## 3. Proposed Method

### 3.1. Overview

In this section, we introduce the STAC network. We use two-stream as the architecture of the proposed model, in which the first stream uses RGB frames as input and the other stream encodes the optical flow. In the two branches, we use the same architecture of CNN-RAN to encode the weight map of deep features from different inputs of RGB frames and optical flow to generate a recurrent attention map. To this end, we developed two benchmark networks: SAC (spatial attention on compensation information) and TAC (temporal attention on compensation information). We use the recurrent neural unit LSTA [20] as our attention tracker on both of the two modules to obtain the local feature of objects and relevant motion flow. We finally combine the output features of the two networks followed by a new fully-connected layer as the classifier to perform fine-grained activity recognition from a weakly supervised setting. The overall framework is illustrated in Figure 2.

### 3.2. SAC Module

#### 3.2.1. Size Compensation

As the camera is fixed on the head, the size of the objects visible in FPV is almost single-scale and smaller than the size of the objects in ImageNet. In addition, the objects are fine-grained which are less in the same category. The problem makes it is difficult to identify activate objects. Thus, we compensate for the data in the aspects of scale and size. We use random horizontal flipping and multi-scale corner clipping techniques proposed by Li et al. [11] for data augmentation and size compensation. The original frames are scaled to 340×256 to appropriately reduce the amount of computation. The augmented sub-images are extracted from the center and four corners of the scaled images with random size ratios from {1, 0.875, 0.75, 0.66}. These multi-scaled regions are then resized to 224 × 224. Then we get a set of local areas containing multi-scaled objects in which some amplified objects are comparable to that in ImageNet.

This operation not only makes the activated features easier to localize but also avoids overfitting. The conclusion is that using local zoomed data leads to more effective performance than simply feeding the entire global information to the network. The multi-scaled regions make the location-aware attention more instructive. This is demonstrated in detail in the experimental comparison in Section 4.3. We used the uniformly sampled 25-frame sequence with equal temporal spacing among the cropped videos as the input of RGB stream, following the test practice in original two-stream ConvNets [9].

#### 3.2.2. Spatial Attention

We use a ResNet-34 pre-trained on ImageNet as the deep feature extractor, following the practice in [30]; the output of the last convolution layer (conv5_x) is extracted as the deep feature for subsequent attention encoding.

The spatial attention mechanism we use comes from LSTA [20]. In order to maintain the potential spatial position information in the convolutional features from being lost in the full-connected layer, they draw on the idea of CAM [15] for its remarkable ability of weakly-supervised fine-grained object localization. The spatial global average pooling (GAP) ϵ(x) is used to generate the video feature descriptor, and then obtain a feature selector that can be trained:

(1) $$c^{*}=argmax_{c}\pi \left(\epsilon \left(x\right),\theta_{c}\right)$$

It can establish a mapping relationship between the category space *C* and the convolution feature planes x with spatial information, and get the category with the highest score, where $\theta_{c}$ represents the score (weights) of x on category *c*, and $c^{*}\in C$ represents category with the highest score. Attention pooling ζ is obtained by a pair of equivalent spatial average pool ϵ(x) and linear mapping $\pi (\epsilon ,\theta_{c})$:

(2) $$\zeta \left(x,\left\{\theta_{c}\right\}\right)=\epsilon^{⊥}\left(\pi \left(x,\theta_{c^{*}}\right)\right)$$

We show a detailed block flow in Figure 2 of deep feature x and the descriptor $c^{*}$, and how the attention pooling ζ works. This pooling operation extends the standard convLSTM. In fact, the training and tracking process of attention map is equivalent to another convLSTM that takes the deep feature x as input. The instance of recurrent training of attention map *M* can be described as:

(3) $$v_{a}=\zeta \left(x_{t},w_{a}\right)$$

(4) $$\left(i_{a},f_{a},s_{t},a\right)=\left(\sigma ,\sigma ,\sigma ,\eta \right)(W_{a}*[v_{a},s_{t-1}⊙\eta (a_{t-1})])$$

(5) $$a_{t}=f_{a}⊙a_{t-1}+i_{a}⊙a$$

(6) $$M=softmax(v_{a}+s_{t}⊙\eta (a_{t}))$$

It can be seen from Equation (3) that at first the deep feature $x_{t}$ and its weight selected by the selector $c^{*}$ (Equation (1)) are used as the input of attention pooling ζ (Equation (2)) to obtain a weight map $v_{a}$ with spatial localization information. Equations (4)–(6) then take this map as the input of a conventional LSTM cell with memory parameter $a_{t}$ and output gate $s_{t}$ whose output is added to the weight map $v_{a}$, followed by a softmax pooling to get the recurrent attention map *M*.

In order to enhance the forget-update ability of the output gate, the input variable of it has been modified. An output pooling built upon the analogy with attention pooling is used to control the output of LSTA as follow:

(7) $$\left(i_{c},f_{c},c\right)=\left(\sigma ,\sigma ,\eta \right)\left(W_{c}*\left[M⊙x_{t},o_{t-1}⊙\eta (c_{t-1})\right]\right)$$

(8) $$c_{t}=f_{c}⊙c_{t-1}+i_{c}⊙c$$

(9) $$v_{c}=\zeta \left(c_{t},w_{c}+w_{o}\epsilon \left(M⊙x_{t}\right)\right)$$

(10) $$o_{t}=\sigma \left(W_{o}*\left[v_{c}⊙c_{t},o_{t-1}⊙\eta (c_{t-1})\right]\right)$$

A standard output of a LSTM takes $M⊙x_{t}$ as the input:

(11) $$o_{t\_s}=\sigma \left(W_{o}*\left[M⊙x_{t},o_{t-1}⊙\eta (c_{t-1})\right]\right)$$

The output of the recurrent process on $v_{a}$ and the process on x are finally coupled by an output gated pooling (Equation (7)) in the form of $M⊙x_{t}$ to enhance the refinement of activation features in the recurrent neural unit and forget the irrelevant information. The output gate (Equation (10)) of LSTA uses $v_{c}⊙c_{t}$ instead of $M⊙x_{t}$ in a standard output gate of LSTM because the current cell state $c_{t}$ (Equation (8)) has integrated the attention filter $M⊙x_{t}$ to maintain the records of historical related areas. $v_{c}$ (Equation (9)) is equivalent to using the attention filter $M⊙x_{t}$ to control the weight bias of the next cell state to generate a discriminative weight map of what to remember. This is the localization of the current activation. The output gate (Equations (9)–(10)) allows the network to cyclically propagate the memory state filtered by attention map *M*. The bold symbols in the equations indicate recurrent variables.

The above formulae of LSTA show that a recurrent attention map *M* enhances the capacity of localizing activate information from the global feature. We address the problem of fine-grained egocentric activity recognition in the RGB stream by establishing a spatial Recurrent location-aware Attention Neural network (RAN) using LSTA to guide the precise localization of objects in the image. Taking the compensated video frames as the input of the RGB stream, we use a ResNet-34 network pre-trained on ImageNet as our backbone. The pre-trained network can direct the recognition of the objects implicitly in the activity recognition task. The deep feature x from the final convolutional layer (conv5_x) and its class activation weight map $v_{a}$ generated by attention pooling ζ are input into the recurrent network for attention circulation and synchronized tracking. The parameters of the other layers of the backbone are fixed and we only fine-tune to the last layer to reduce the number of parameters of our model and make effective use of the pre-trained parameter guidance.

### 3.3. TAC Module

#### 3.3.1. Motion Compensation

**Figure 3:**
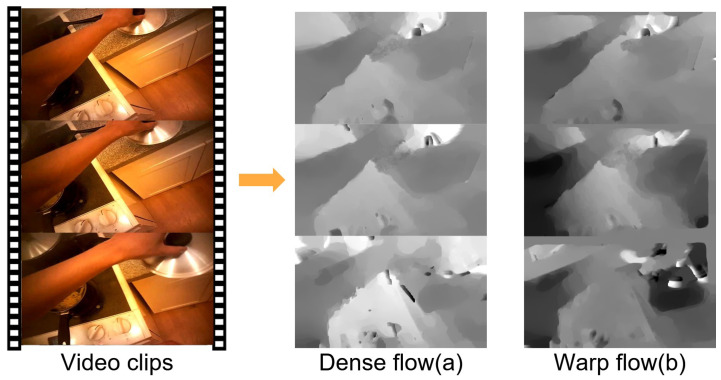
Examples of dense flow (**a**) and warp flow (**b**). Obviously warp optical flow (**b**) suppresses the background movement and highlights the effective motion of the actor more than (**a**).

Due to the normal motion of the wearer’s head, the head-mounted device will introduce additional ego-noise beyond the effective motion to the video, resulting in blurred images and background motion in optical flow. Therefore, camera compensation is very important. Some works [3,11,27,45] compensate for camera motion and have proved their effectiveness in action recognition. We use the method proposed by Wang et al. [27] to make compensation to flow fields. The homographic matrix is estimated through RANSAC feature matching between two consecutive frames and the second frame is warped by this matrix. According to this, the optical flow can be recalculated between the first and the warped second frame. Then the trajectories from camera motion are compensated. Figure 3 provides the comparison between dense flow (a) and the compensated warp flow (b) which shows the obvious removal of background motion. The warp optical flow is using as the input modality to the motion stream and a remarkable improvement is achieved by this. We demonstrate this in Section 4.3.

#### 3.3.2. Temporal Attention

In order to make up for the lack of training data, pre-training has become an effective method for initializing deep ConvNet [9]. The fine-grained characteristic and long-tail nature of egocentric datasets are even worse. The pre-trained ResNet-34 network on ImageNet uses RGB frames as input which is different from the distribution of the optical flow input modality. In order to use the pre-trained network in temporal sequence, we follow the cross-modal initialization method proposed by Li et al. [11]. The pre-trained backbone on RGB is modified by averaging the weights on its first convolutional layer and replicating them according to the number of input channels in the motion stream to initialize the temporal backbone network.

We first train the verbs in egocentric activities (such as “stir”, “wash” and “cut”) using the modified ResNet-34 pre-trained on ImageNet. Location-aware channel attention similar to the SAC network is deployed to the TAC network in the way of stacked optical flow. The pre-trained weights on verbs are loaded to initialize the TAC net.

Inspired by [46] in which a mid-layer fusion is proposed to ensure the channel responses at the same pixel position of spatial and temporal information are put in correspondence, we make a channel correspondence between temporal and spatial stream. (1) In order to let LSTA have the ability to localize activated features on temporal stream, we follow the practice in original two-stream ConvNet [9] to stack the warp optical flow channels $d_{t}^{x,y}$ corresponding to 5 consecutive frames in the *x* direction and *y* direction as the input of the TAC network. The size of the TAC input tensor is [5,10,224,224] which has the same dimension as the size of input tensor [25,3,224,224] of the SAC. The former has a total of 5(frames)×2(directions) channels, which is analogous to the 3 channels in RGB frames. 25 indicates the sequence length of the input in RGB stream. In addition, the original short-term video clips are not specifically designed to crop according to the task time and some activities have a long duration. These videos are recorded in real daily activities. As a consequence, relevant frames may appear anywhere in the video clips. We address this problem by evenly sampling 5 groups of 5 consecutive frames of flow fields located in the time series. (2) We extract the final convolution layer of ResNet as temporal deep feature map, same as the practice in RGB stream, and use spatial location-aware attention to encode the temporal feature. So the TAC module can filter the features of the stacked temporal channels, so as to track the effective motion related to the recognition task. STAC which is the fusion of SAC module and TAC module then has the ability to combine spatial and temporal features.

## 4. Experiments

In this section, we first introduce the details of the datasets and the experimental settings and the evaluation methodology we used. We report the significant improvement of size compensation and motion compensation through extensive ablation analysis, explore the strong guidance of location-aware attention in the task of egocentric activity recognition. The attention maps generated by our model are visualized below to give an intuitive explanation. The validity of the proposed model is verified by comparison with state-of-the-art technology.

### 4.1. Datasets and Validation Methodology

We verify the effectiveness of our method on two challenging datasets GTEA [3] and EGTEA_GAZE+ [5] available on http://cbs.ic.gatech.edu/fpv/. GTEA_61 is a relatively small dataset that contains 61 classes of daily activities. It is suitable for extensive ablation analysis to verify the effectiveness and necessity of each part of the model on the premise of saving time and equipment resources. An ablation study was conducted on a fixed split (S2) of GTEA_61 and the quantitative results are shown in Section 4.3. EGTEA_GAZE+ is a large-scale self-centered video dataset, containing 10,325 fine-grained action instances, including 19 verb categories, 51 noun categories and 106 activity categories. We use the leave-one-subject-out cross-validation as the evaluation methodology to conduct the training of the three train-test splits provided by the official datasets and report the accuracy of activity classification on the unseen test sets.

### 4.2. Experiment Settings

At first, The SAC and TAC networks are trained independently to minimize the corresponding cross-entropy losses $L_{RGB}$ and $L_{Flow}$. Then the output vectors of the two streams are combined and we fine-tune the whole model up to the final convolution layer in an end-to-end manner to minimize the sum of the $L_{RGB}$ and $L_{Flow}$. The two branches both use a corresponding ResNet-34 as the backbone that initialized with pre-trained weights on ImageNet. In practice, we compared ResNet-34 and ResNet-50, found that in this task, there is no significant difference in the performance between the two nets. The structure of ResNet-50 is a bit redundant, so we choose ResNet-34 as our backbone. SAC and TAC both follow a two-stage training strategy.

For the SAC network, we follow the training strategy used in [20]. In the first stage, we first train the LSTA cell and the classifier of SAC. In the second stage, we fine-tune the last convolutional layer and the full-connected layer of the ResNet-34 network to get the inputs of the attention pooling (indicated in the orange blocks in Figure 2). Additionally, the two modules that trained on stage 1 are fine-tuned taking the deep feature and its weight map as input. We use the Adam optimization algorithm with a weight decay of 0.0005 for training.

For the TAC network, we first use the initialization method mentioned in Section 3.3.1 to obtain a ResNet-34 suitable for optical flow as input, use it to pre-train the actions (the action in an activity category refers verb, such as “take”, “put”, “pour” and “stir”). Then the TAC net is initialized with the weights trained on action verbs and fine-tuned from layer conv5_3 of the ResNet-34 network, same as the practice in stage 2 of the SAC network. In the training of Flow, the SGD algorithm with the momentum of 0.9 and weight decay of 0.0005 ($L^{2}$ norm regularization) is used as an optimizer.

After obtaining the above two pre-trained branches, we perform end-to-end two-stream fusion training by concatenating the output tensors of the two networks together followed by a fully-connected layer for classification. The fine-tuning strategy of the two-stream network is still the same as stage 2 of the above two networks that the model is trained down to the last convolutional layer of the CNN. Table 1 shows the hyperparameter settings on different datasets during training. Among them, the parameters of SAC and TAC are given in the form of “stage1/stage2”; “Learning-rate” refers to the initial learning rate and “Decay-rate” represents the decay multiple of the learning rate. If the “Decay-step” is 1, it means the learning rate is decayed at the corresponding multiple of each epoch; while the form of a list (such as [50,75]) indicates a decay after the corresponding epoch in the list. The LSTA in the network has 512 hidden units. “num_classes” ($w_{c}$-Equation (9)) represents the number of categories in output pooling, following the setting in [20], is selected appropriately according to the number of activity categories in the datasets.

### 4.3. Extensive Ablation Study

We conduct extensive ablation studies on the fixed split (S2) of the GTEA_61 dataset to evaluate the effectiveness of each component in STAC. The proposed model consists of two CNN-RAN branches with the same structure, each branch contains (1) input augmentation and compensation, (2) backbone module for deep feature extraction and (3) recurrent attention neural network. Table 2 shows the comparison between baseline and different modules with each component on the RGB stream network SAC and the motion stream network TAC respectively. Additionally, the performances after joint training of the above two branches are reported. We follow the practice in the LRCN approach proposed by Donahue et al. [30] to extract the last convolutional layer of the CNN as a deep hierarchical visual feature descriptor.

#### 4.3.1. SAC-RGB

**Figure 4:**
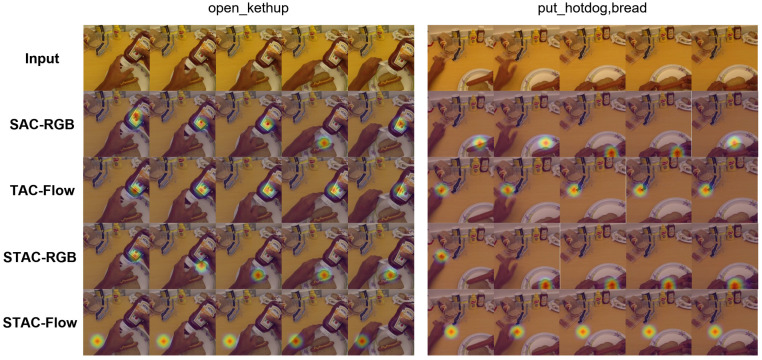
Attention maps generated by our model. We chose 5 frames uniformly sampled from the input of SAC and we visualize the attention map generated by TAC on these frames using the stack of corresponding warp flow as input. The first row shows cropped raw frames; the second and third rows show the attention map generated by our SAC and TAC independently. The fourth and fifth rows correspond to SAC and TAC after two-stream joint learning. We present two groups of attention maps corresponding to two activities.

The first section in Table 2 gives a detailed description of extensive experiments of RGB stream. (1) We choose ResNet34-convLSTM as the baseline. (2) SA-RGB (refers to non-compensated input + backbone + Spatial Attention) takes the uniformly sampled 25-frame sequence of the original cropped videos as input. The input frames are not compensated in size. We randomly crop regions of 224×224 size from the frames as the input of CNN. We replace the RNN (LSTM) in the baseline with LSTA, which can preserve the spatial position information and smoothly track the activation regions. The injection of spatial position-aware attention brings the model 9% improvement compared to the baseline. Compared with LSTM, which only tracks the convolutional features, LSTA firstly screens the deep features to get spatial attention of activated information, and then tracks the attention and suppresses irrelevant information. (3) SAC-RGB (refers to compensated input + backbone + Spatial Attention) compensates for the input frames at first. The original frames are scaled to 340×256 to appropriately reduce the amount of computation. We use the random horizontal flipping and multi-scale corner clipping techniques introduced in Section 3.2.1 to compensate for the single-scaled small-sized fine-grained objects in FPV. Another 9% improvement is observed compared to SA-RGB. Compared to the baseline, our SAC module on the activity prediction significantly improves the accuracy from 51.69% to 70.69%. The second and fourth rows in Figure 4 show the attention map generated by the SAC network independently and after joint with TAC respectively. It can be seen that the network pays attention to the object under operation.

#### 4.3.2. TAC-Flow

The second section in Table 2 shows the detailed comparison of extensive experiments of Flow stream. (1) To verify the importance of camera compensation, we first conduct a comparative experiment on the modified ResNet-34 (baseline of Flow stream) mentioned in Section 3.3.2 with different optical flow as input. (2) TC-Flow (refers to Temporal Compensation module) takes the stacked warp optical flow as input as mentioned in Section 3.3.1, while the comparison baseline uses stacked dense flow as input of the modified ResNet-34. It can be seen from Table 2 that the TC-Flow network with warp flow as input obtains a 3.5% performance improvement, as a consequence, the camera motion was compensated. Hence, the stacked warp optical flow is used as the input modality of the TAC network. (3) We build temporal location-aware channel attention upon the analogy with LSTA in SAC, to get more accurate localization of the effective motion. An accuracy of 51.72% is obtained by TAC net which is 7% higher than the baseline.

The third and fifth rows of Figure 4 show the visualization results of the attention maps generated by the SAC network independently and after joint with TAC respectively, on the 5 RGB frames corresponding to the flow input. It can be seen that the application of attention in the temporal sequence can well track the motion trajectory and locate the position where the movement occurs, which is mainly concentrated around the hands. It is complementary to the attention of the appearance stream which concentrates on the object area.

#### 4.3.3. STAC

We fuse the above two pre-trained single-stream networks and perform end-to-end training of spatiotemporal information to obtain the STAC network. The network performance has been improved by a significant margin (SAC + 10% and TAC + 30%) after two-stream joint training, reaching 80.17%. The significant improvement from SAC and TAC to STAC intuitively proves the complementary relationship between the two modules. We will explain this further in terms of the discussion of visualization results in Section 4.5.

We also present the importance and differentiating factor of STAC compared to STC and TAC by verifying the ability of the three networks to recognize verbs and nouns in Table 3. (1) It is obvious that the noun recognition accuracy in SAC is higher than in TAC by a significant margin (+24%), while the verb recognition ability of TAC is better than SAC (+2%). This echoes the above argument that spatial attention screen the activated objects and temporal attention focuses on effective motion. (2) After two-stream joint training, the accuracy of verbs and nouns has been greatly improved. The verb recognition accuracy of the STAC model is 7% higher than that of the TAC. This is because the object location information of the appearance stream provides a prior information for the attention of the motion. Additionally, the noun recognition accuracy of the STAC model is 6% higher than that of the SAC, indicating that the effective motion trajectory in the optical flow has made an addition to the localization of the object. (3) Besides, we observe that the performance of nouns prediction accuracy is lower than that of verbs in SAC (77.59% compared to 82.67%) and STAC (83.62% compared to 91.38%) with the input of RGB frames, which is caused by the fine-grained objects in FPV. Action categories are much less than object categories. To further improve the ability of noun recognition may require strong supervision of localization information such as bounding boxes.

### 4.4. Comparison with State-of-the-Art Results

We verified the superiority of our method on GTEA_61 and a large-scale dataset EGTEA_GAZE+ by comparing with the following state-of-the-art technology. The first part of the Table 4 including 3 mature action recognition methods mainly designed for third-person video in which TSN [11] achieves the best results. The methods listed in the second part of Table 4 use strong supervision in the training phase. EgoIDT [3] needs hand segmentation and gaze information, and their follow-up work [5] also use the gaze information for attention encoding. SAP [35] takes the faster R-CNN as a third stream to extract prior information which requires bounding box annotations. However, as argued in Section 4.3, it is undeniable that object-level detail information plays a very important role in first-person video analysis because the objects in FPV images are smaller and are fine-grained targets compared to those in third-person images. This method currently has the best performance. The two models in the third part are from the same author both using the CNN-RNN structure. The previous work ego-rnn [17] takes the softmax calibrated CAM map as spatial attention which then inputs to LSTM. They apply attention only in the RGB stream to focus on objects. Their subsequent work LSTA [20] embedded an attention pooling into the LSTM. The LSTA is a novel recurrent neural unit and the attention maps are used as a filter of activation in the process of forget-update.

We take LSTA to establish the RAN unit in both the RGB stream and the temporal stream to conduct spatial-temporal attention modeling and fine-grained activity recognition. Our model STAC outperforms the state-of-the-art performance on all the three train-test splits of the EGTEA_GAZE+ dataset. The RGB stream achieves an average accuracy of 60.45% on the unseen test sets. Considering the complementarity of optical flow information, we generate attention on warp optical flow stacks to localize motion features. The TAC achieves an average accuracy of 46.03% only with the optical flow as input. We conduct synchronization training of STAC after the fusion of the above two branches for the final activity classification whose performance improved by a large margin (+3% from SAC and +17% from TAC). The significant improvement mainly benefits from the compensation information and spatial-temporal attention. Compared to LSTA [20] which also utilizes the two-stream CNN-RNN structure, our method outperforms by 1.6%. Compared with the SAP which uses the third stream as privileged information, we obtain a 0.85% improvement.

### 4.5. Discussion

#### 4.5.1. Visualization

Figure 4 shows two groups of attention maps for different activities, which qualitatively reflect the complementary characteristics of SAC and TAC models, indicating that attention is important in the temporal stream as it in RGB stream. We use CAM [15] to visualize the classification results. Spatial attention from SAC (second row) is more sensitive to the object being manipulated, while temporal attention from TAC (third row) focuses obviously where the movement occurs. The attention maps of the two networks after joint training have obtained more precise localization; see fourth and fifth rows. In activity 1 (“open_kethup”), STAC-RGB emphasizes the cap instead of the bottle, while STAC-Flow changes the attention from the bottle to hand. It suggests the two benchmarks are complementary. The prediction labels of the corresponding confusion matrix bellow also prove this point.

The comparative activity group (“put_hotdog, bread”) shows that among the activity with large displacements of motion, the jointly trained STAC-RGB has a more obvious improvement than the independently trained SAC. The frame 1 (column 6, row 4 compare to row 2) of “put_hotdog” shows the attention shifts follow the actor’s sight from “hotdog” on the “bread” below the frame to “hotdog” bag above. While temporal attention only works for frame 1 and frame 2. From the input frames in the first row, we found that the follow-up activity occurs at the bottom of the field of view so that the TAC does not fully capture the motion. This is because the video is acquired by the camera worn on the head, and the sight of the camera is higher than that of the actor resulting in the lack of subsequent optical flow information. In conclusion, the transition of the attention from second (third) row to fourth (fifth) row suggests the effectiveness and complementary of spatial-temporal location-aware attention.

#### 4.5.2. Macroscopic Result

**Figure 5:**
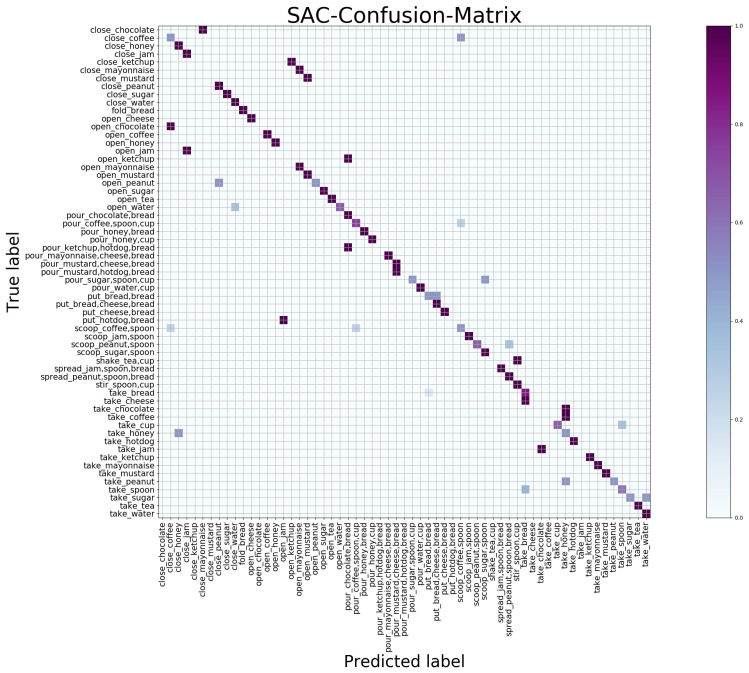
Confusion matrix of SAC generated by independent training on GETA_61 dataset.

**Figure 6:**
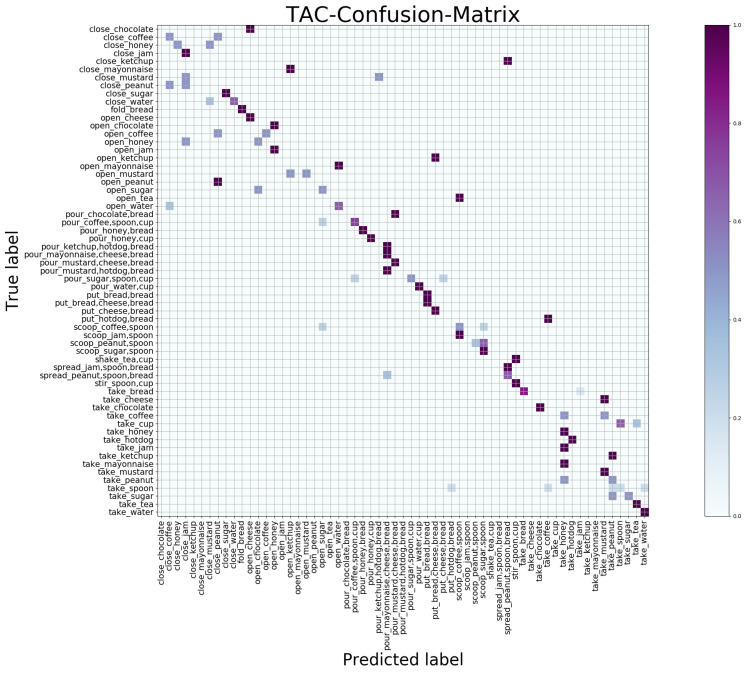
Confusion matrix of SAC generated by independent training on GETA_61 dataset.

**Figure 7:**
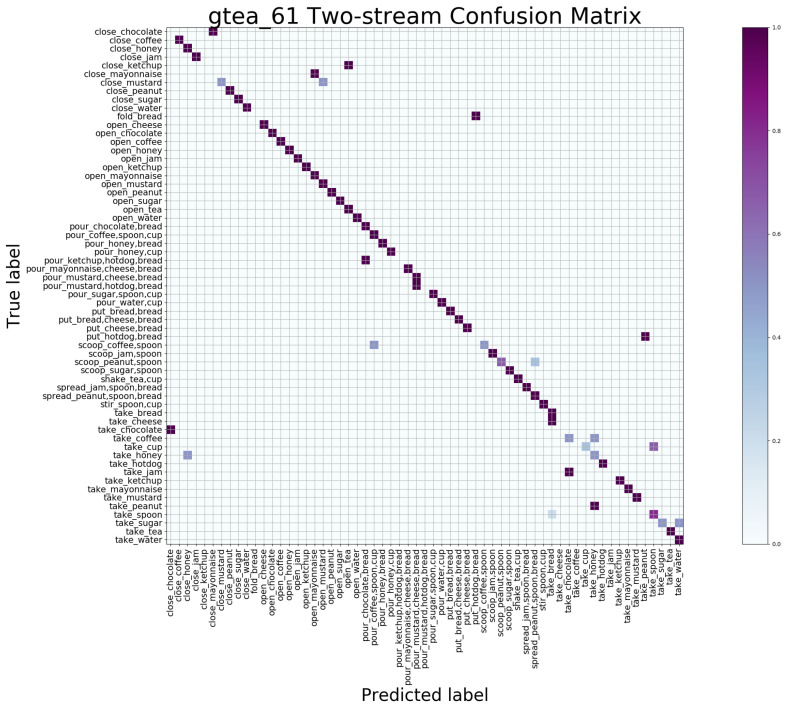
Confusion matrix of STAC generated by joint training on GETA_61 dataset.

The confusion matrix is a standard format for accuracy evaluation which can observe the performance of the model in each category. We give the confusion matrix maps obtained by the SAC and TAC network trained separately in Figure 5 and Figure 6 and the results of jointly trained STAC in Figure 7, showing the macroscopic results of the egocentric fine-grained activity prediction on GTEA_61 dataset qualitatively and quantitatively. The positive values of the prediction are on the diagonal, while the negative values should be outside the diagonal. The *x* axis is the predicted category label, and the *y* axis is true category label.

By observing the predicted label, we found that in the SAC network Figure 5 taking RGB frames as input, the recognition of the objects is better than that in the TAC network Figure 6, but the action prediction results are worse due to the lack of motion information. This conclusion has been quantitatively explained in Table 3. It is obvious from the label of prediction that, for example, in Figure 5, “close_chocolate” or “open_chocolate” has been predicted as “pour_chocolate”; “open_cheese” has been predicted as “put_cheese,” suggesting that the motion information is important for verb recognition.

In the case of optical flow information as input, the TAC network lacks object appearance information. In Figure 6, the predicted negative values tend to cluster in several categories. This is obvious in the upper left and lower right corners of the TAC-confusion-matrix map (visually tends to be on several vertical lines); that is, different activity classes with the same motion (verb) are easy to be predicted as the same activity category (such as “close” and “take”). Additionally, the SAC branch compensates for the lack of location information of objects exactly. This kind of complementary relationship between SAC and TAC can also be seen in the attention map in Figure 4. We have given a detailed argument based on the attention map above.

In Figure 7, significant positive aggregation can be observed, indicating the complementary effects of the two branches. Our STAC model makes full use of appearance information and optical flow information, and hence obtains accurate localization of objects and effective capture of motion with the help of spatial-temporal attention. We analyzed the negative values after the two-stream union and found that the predicted results of some verbs (e.g., “close” and “open”) are easily confused; this is caused by the similarity of the motions, and objects with similar appearance are also prone to prediction failure, such as “mayonnaise” and “chocolate sauce” as they are both bottled sauces.

**Figure 8:**
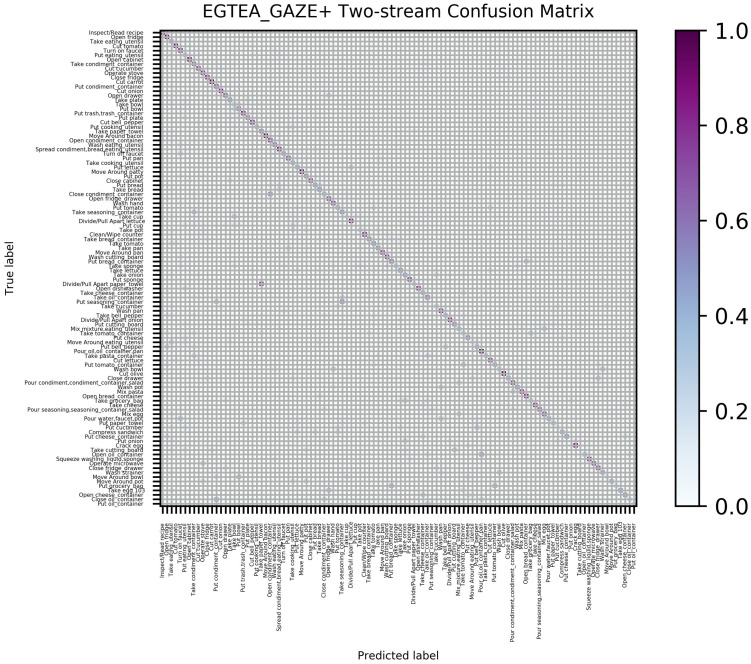
Average confusion matrix of three training-set splits generated by STAC on EGETA_GAZE+ dataset.

For the large dataset EGTEA_GAZE+, the confusion matrix of jointly training is shown in Figure 8. We mainly discuss the negative values of the predicted results. The negative values are mostly distributed at the lower diagonal; that is, the activity categories of the tail (fewer data for training) will be predicted as the category of the front part (more data for training), which is caused by the long tail characteristic of the large dataset. In other words, there are a variety of egocentric activities; the activities in the front of the data list may appear frequently in daily life, while the latter activities have a low frequency of repetition, leading to data imbalance. Activity categories with less training data have poorer prediction results and will be mistaken for the categories with more data seen by machines.

## 5. Conclusions and Future Work

In this work, we present a generic spatial-temporal attention network based on compensation information to recognize egocentric activities in a weakly supervised environment. Our model STAC is a two-stream CNN-RAN structure, applying location-aware attention to both branches. The RAN is a recurrent neural unit with the ability to generate recurrent attention maps that maintain the activation. The size-compensated RGB sequence and the motion-compensated warped optical flow stacks are encoded separately by spatial position attention and temporal channel attention, which are then synchronously coupled with the deep features to screen the effective information. The extensive experiments have proved the effectiveness of our framework, and our model outperformed the state-of-the-art methods. We conducted extended ablation analysis on GETA_61 dataset, including 61 activities to further illustrate the necessity of compensation and the guiding role of spatial-temporal location-aware attention for activity recognition in FPV. As future work, we hope to interpret the tracked attention in such a way that the visualized transfer sequence will be able to locate multiple objects in a long-term task. 

## Figures and Tables

**Table 1:** The parameters of the experiments corresponding to the two datasets.

Dataset	GTEA_61 (Stage1/Stage2)			EGTEA_GAZE+ (Stage1/Stage2)		
Parameter	SAC	TAC	STAC	SAC	TAC	STAC
epoch	200/100	700/500	200	200/100	350/300	200
Learning-rate	1 × $10^{-3}$/1 × $10^{-4}$	1 × $10^{-2}$/1 × $10^{-2}$	1 × $10^{-3}$	1 × $10^{-2}$/1 × $10^{-4}$	6 × $10^{-3}$/1 × $10^{-3}$	1 × $10^{-3}$
Decay-rate	0.1/0.1	0.5/0.5	0.99	0.1/0.1	0.1/0.5	0.99
Decay-step	[25,75,150]/	[75,150,250,500]/	1	[25,50,75,100]/	[50,75,100,150]/	1
	[25,75]	[25,75,150,300]		[10,25,50,75]	[25 50 100]	
LSTA-depth	512			512		
Num-classes	100			150		

**Table 2:** The Ablation analysis of our methods on the GTEA_61 dataset.

	Methods	Performance (%) on Fixed Split (S2)
	Baseline(RGB)	51.69
	SA-RGB	61.21
Extensive	**SAC-RGB**	**70.69**
ablation	Baseline(Flow)	44.75
study	TC-Flow	48.28
	**TAC-Flow**	**51.72**
	**STAC**	**80.17**

**Table 3:** Comparison of recognition accuracies of verbs and nouns with that of activities.

Model	Verb	Noun	Activity
SAC	82.76	77.59	70.69
TAC	84.48	53.45	51.72
STAC	91.38	83.62	80.17

**Table 4:** The comparison with the state-of-the-art methods on the GTEA_61 and EGTEA_GAZE+ datasets.

Methods	GTEA_61 (%)	GTEA_GAZE+ (%)			
	Fixed Split	Split1	Split2	Split3	Cross Valid
Two Stream [9]	57.64	43.78	41.47	40.28	41.84
I3D [10]	/	54.19	51.45	49.41	51.68
TSN [11]	67.76	58.01	55.01	54.78	55.93
EgoIDT [3]	66.8	/	/	/	46.5
Li et al. [5]	/	/	/	/	53.3
SAP [35]	/	64.1	62.1	62.0	62.7
Ego-rnn [17]	77.59	62.17	61.47	58.63	60.76
LSTA [20]	79.31	/	/	/	61.86
**SAC-RGB**	**70.69**	**62.69**	**60.20**	**58.47**	**60.45**
**TAC-Flow**	**51.72**	**47.23**	**46.44**	**44.43**	**46.03**
**STAC**	**80.17**	**64.41**	**63.17**	**62.86**	**63.46**

## Data Availability

Publicly available datasets were analyzed in this study. This data can be found here: [http://www.cbi.gatech.edu/fpv/].

## Abbreviations

The following abbreviations are used in this manuscript:

RANSAC	RANdom SAmple Consensus
Adam	Adaptive moment estimation
SGD	Stochastic Gradient Descent
